# Infant with right hemiplegia due to acute encephalopathy with biphasic seizures and late reduced diffusion (AESD): A case report

**DOI:** 10.1097/MD.0000000000025468

**Published:** 2021-06-04

**Authors:** Ai Takahashi, Erina Kamei, Yuri Sato, Seiichiro Shimada, Misao Tsubokawa, Genrei Ohta, Yusei Ohshima, Akihiko Matsumine

**Affiliations:** a Division of Rehabilitation Medicine, University of Fukui Hospital; b Department of Orthopaedics and Rehabilitation Medicine; c Department of Pediatrics, University of Fukui, Fukui Prefecture, Japan.

**Keywords:** acute encephalopathy with biphasic seizures and late reduced diffusion, case report, functional recovery, hemiplegia in children

## Abstract

**Rationale:**

Acute encephalopathy with biphasic seizures and late reduced diffusion (AESD) is a condition characterized by biphasic convulsions and disturbance of consciousness. In Japan, the most common pediatric cases of acute encephalopathy are associated with infection. AESD usually occurs in early childhood, with the characteristic magnetic resonance imaging (MRI) appearance called “bright tree appearance.” The disease often has neurological sequelae and interferes with the schooling of children and their activities of daily living; however, there are few clinical case reports of hemiplegia caused by AESD.

**Patient concerns:**

A case with right-sided hemiplegia due to AESD in an 11-month-old girl who was followed up to 30 mo of age.

**Diagnoses:**

The patient was diagnosed with overlap AESD and hemiconvulsion-hemiplegia-epilepsy syndrome (HHE syndrome), based on the clinical course and imaging findings. DNA tests of her blood and cerebrospinal fluid revealed the presence of human herpesvirus 6.

**Interventions:**

Pharmacotherapy and rehabilitation therapy.

**Outcome:**

Gross motor function has recovered considerably, but she had a mild developmental delay at 30 mo old.

**Lessons:**

Hemiplegia due to AESD was extremely rare, and appropriate rehabilitation treatment resulted in recovery of physical function. However, as mild developmental delay was observed, the patient was referred to a specialized facility before entering school.

## 1 Introduction

Acute encephalopathy with biphasic seizures and late reduced diffusion (AESD) is a condition characterized by biphasic convulsions and impaired consciousness, triggered by viral infections. It accounts for the majority (29%) of cases of acute pediatric encephalopathy in Japan. Approximately 2000 to 7800 new patients are diagnosed annually, and it is designated as an intractable disease under the Intractable Diseases Law.^[1,2]^ It frequently occurs in children from 6 mo to 1 yr of age. In a typical case, a prolonged febrile seizure (early seizure) occurs on days 1 to 2, followed by a cluster of complex partial seizures (late seizures) on days 3 to 7.^[3]^ Brain magnetic resonance imaging (MRI) performed within 2 d of the initial prolonged seizure shows no abnormality. However, between days 3 and 9, high-signal intensity is observed in the subcortical white matter on the diffusion-weighted image (DWI), which is called the “bright tree appearance (BTA).”^[4]^

The disease is sometimes misdiagnosed as febrile seizures in the early phase. Febrile seizures are the most common convulsive disorders in children with fever. The risk factors for developing this disease (sensitivity 88.7%, specificity 90%).^[5]^ Here, we report a case in which biphasic convulsions led to right hemiplegia. Although the motor function recovered, mild intellectual disability was noted. We also considered this case as the overlap of AESD and hemiconvulsion-hemiplegia-epilepsy syndrome (HHE syndrome).^[6]^

## 2 Case report

Written informed consent was obtained from the guardians of the participant before the beginning of the study. The patient was a healthy 11-month-old girl. Her weight at birth was 4080 g, and she was delivered by vaginal birth at 40 wk and day 0 of gestation. Her development was normal. She received vaccinations designated by the government and did not have any allergies. She learned to roll over, sit up, and crawl during her development. Two weeks before the first consultation, her body temperature was 100°F (37.8°C) or higher intermittently, and her family doctor prescribed antipyretics. On the day of consultation at our hospital, she had a fever of 102.2°F (39.0°C), and she had her first seizure, which was of tonic-clonic type. Twenty-four minutes after the onset of convulsion, she was transported to our hospital, and the seizures subsided 40 minutes after the initial onset, after inserting a diazepam suppository and intramuscular injection of midazolam. We temporarily intubated her for respiratory management, and she was extubated the next morning due to recovery of spontaneous breathing.

## 3 Assessments

The blood reports at the time of the consultation are shown in Table 1.

**Table 1:** Blood analysis at first consultation.

Blood count (reference range)				Blood biochemistry			
WBC	39.0	×10^3^ μL	(4.3-19.1)	AST	60	U/L	(24–57)
RBC	4.62	×10^6^ μL	(3,93–5.38)	ALT	29	U/L	(9–38)
Hb	11.6	g/dl	(10.7–14.1)	LDH	524	U/L	(202–437)
Hct	38.8	%	(31.7–42.4)	CK	81	U/L	(39–295)
Plt	504	×10^3^ μL	(168–650)	TP	6.8	g/dl	(5.7–7.5)
				BUN	12	mg/dl	(3.7–18.6)
Venous blood gas analysis				Cr	0.33	mg/dl	(0.3–0.6)
pH	6.881			Na	131	mEq/L	(135–143)
pCO_2_	124.0	mm Hg		K	5.0	mEq/L	(3.6–5.1)
pO_2_	52.1	mm Hg		Cl	101	mEq/L	(101–110)
HCO_3_	22.1	mmoL/L		Ca	8.9	mg/dl	(8.8–10.6)
B.E.	−15.0	mmoL/L		Glu	294	mg/dl	
				NH_3_	46	μmoL/L	
				CRP	0.06	mg/dl	(0.045–12)

**Figure 1:**
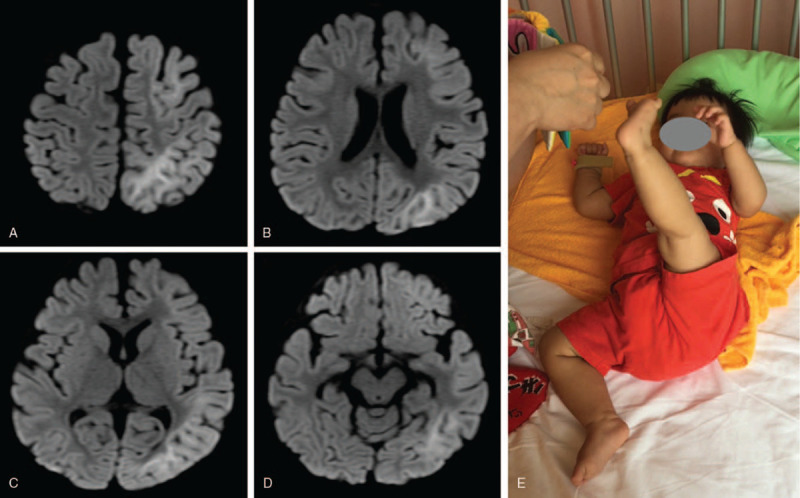
MRI and physical findings of the patient. A-D: Brain MRI on day 4. BTA pattern was seen in the left cerebral hemisphere on DWI. E: Photograph at the start of rehabilitation. The patient presented severe right hemiplegia. The response to the toy was good. BTA = bright tree appearance, DWI = diffusion-weighted image, MRI = magnetic resonance imaging.

The reports showed a high leukocyte count of 3.9 × 10^3^ μL (reference range; 4.3–19.1), aspartate aminotransferase (AST) 60 U/L (reference range; 24–57), alanine aminotransferase (ALT) 29 U/L (reference range; 9–38), and blood glucose 294 mg/dl. She had a complex partial seizure only in the right half of the body (second seizure) on day 3, followed by 4 d of intermittent seizures. The BTA pattern was noted on brain MRI on day 4 in the subcortical white matter of the left cerebral hemisphere on DWI [Fig. 1 (A-D)].

We diagnosed her as a case of AESD, not febrile seizures, based on the clinical course and imaging findings and initiated anticonvulsant therapy. DNA tests of her blood and cerebrospinal fluid revealed the presence of human herpesvirus 6; hence, we administered prednisolone pulse therapy and intravenous immunoglobulin therapy as well as intravenous antiepileptic drugs. No seizures occurred after day 7, and we transferred her to the rehabilitation department on day 13. Physical findings included frog limb position, gripping of fingers, increased deep tendon reflex, and decreased parachute reflex in the right half of the body, indicative of right hemiplegia. Brunnstrom recovery stage II was recorded in the upper limb and finger, and stage I in the lower limb. Her hand function showed poor passive assist according to the House functional classification system.^[7]^ On the other hand, she was able to communicate almost normally; cried cheerfully, laughed aloud, and babbled spontaneously. (Fig. 1-E).

**Figure 2:**
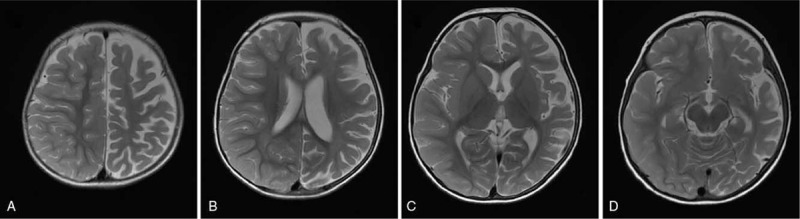
MRI findings 12 months after the onset. A-D: T2-weighted MRI showed atrophy of the left hemisphere.

Physical therapy and occupational therapy were initiated on day 14. During the rehabilitation program, we encouraged a range of motion exercises of the right side, playing with both hands, and sitting practice to prevent retraction of the shoulder girdle and stabilize the sitting position. Initially, she did not use the right limbs at all and fell to the paralyzed side easily. Home rehabilitation programs were continued after discharge on day 23. Her parents enthusiastically trained her and reported to us her state at home. There were no adverse events related to rehabilitation therapy. Five weeks after the onset, automatic movement of her right limbs increased, and she was able to sit alone and turn to the paralyzed side. At 7 wk from onset, she was able to roll over to both sides. After the sitting position stabilized, we encouraged her to stand while supporting her trunk. Six months after the onset, she began to pull herself up at 18 mo of age. At 24 mo, she could walk independently. Simultaneously, there was an improvement in the movements of her right hand; however, skilled movement was performed mostly using the left hand. Brain MRI at 12 mo after the onset showed atrophy of the left hemisphere (Fig. 2).

**Figure 3:**
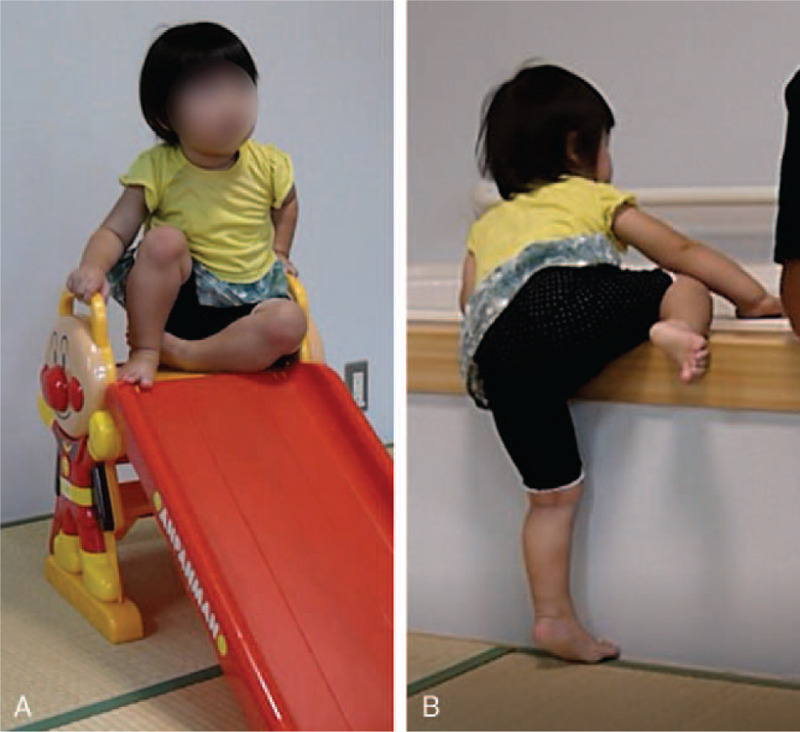
Physical function at 30 months old. A: The patient was able to play on a slide, grabbing the handrail with her right hand. B: Using the paralyzed side, she climbed into the tub (in the training room).

At 28 mo of age, her skills were somewhat delayed compared with other children of the same age at the nursery school. On the Enjoji Infantile Developmental Scale,^[8]^ one of the major pediatric development scales used in Japan, her score at 19 mo of age was a normal score for 14 mo-old children, and at 32 mo of age she achieved a score that is normal for a 20 mo-old. At 29 mo, we evaluated her using the Kyoto Scale of Psychological Development,^[9]^ widely used by Japanese clinicians. The overall developmental quotient (DQ) was 71, Physical-Motor DQ was 51, Cognitive-Adaptive DQ was 76, and Language-Social DQ was 68. These results indicated that she had a mild developmental delay in all items. At 30 mo old, she was referred to a specialized facility for further specialized nursing support and future schooling. Before the referral, her gross motor function had recovered to the extent where she could play on infant slides and climb into the home tub using the paralyzed side (Fig. 3).

## 4 Discussion

There are few clinical case reports of hemiplegia caused by AESD. We described a case of hemiplegia due to AESD in an 11-month-old girl who was followed up for more than a year and discussed the functional recovery.

Acute encephalopathy in children is a syndrome with cerebral edema and accompanying symptoms, such as disturbance of consciousness, convulsions, and abnormal behavior, and is often triggered by infections with human herpes virus, influenza virus, rotavirus, and the like. AESD was first reported by Maegaki et al and Okamoto et al.^[10,11]^ It has been frequently reported in East Asia, and the number of new cases diagnosed in Japan is 100 to 200 per year.^[1]^

The disease typically causes initial convulsions and disturbance of consciousness after fever, following which the patient regains consciousness until convulsions reappear on days 3 to 7 after the onset.^[3]^ MRI findings in the early stage after the onset are usually normal; however, DWI on days 3 to 9 show BTA in the subcortical white matter.^[4]^ This case was extremely rare because it presented with hemiplegia after biphasic seizures, combining the characteristics of both AESD and HHE syndrome. Control of convulsive seizures is an important part of the treatment and might improve the prognosis by reducing the degree of neuronal damage caused by toxicity due to neuronal excitation during status epilepticus. In addition to antiepileptic drugs, the usefulness of steroid pulse therapy, anti-cytokine therapy, and gamma globulin preparations has been reported.^[3]^

A previous study indicated that 66.2% of patients with this disease have mild or moderate sequelae, and 25.1% have severe sequelae.^[2]^ According to the Pediatric Cerebral Performance Category scale,^[12]^ the patients categorized as having moderate disability require special education, and those categorized as having severe disability are not able to attend school and need daily support because of impaired brain function. On the other hand, in terms of motor function, hemiplegia is classified as the least severe (level I) according to the Gross Motor Function Classification System,^[13]^ and it is predicted that independent walking can be acquired at the age of 2. There are several reports about the plasticity of brain function, and it has been reported that hemiplegic patients with childhood-onset have better motor function recovery than adults,^[14]^ and that both hemispheres are involved in the long-term recovery of language function.^[15]^ In this case, 12 months of developmental delay was observed at 30 months of age; however, since the onset occurred in infancy, the recovery from paralysis was good, and future development of language function is expected.

The rehabilitation approach to pediatric hemiplegic patients is stepwise and depends on the recovery from paralysis and developmental aspects. Initially, our patient was unable to sit without support and tilted toward the paralyzed side. In the first phase of the program, the patient was trained to sit while her trunk was supported by the trainer's hand. Another method of correcting the inclination is placing a cushion on the seat on the paralyzed side. After the sitting position stabilized, gait training was initiated, with the trunk held by trainers or using a normal baby walker. For upper limb training for hemiplegic patients, constraint-induced movement therapy is recommended. This type of therapy consists of restraining the unaffected side and increasing the frequency of use of the paralyzed side.^[16]^ However, recently bilateral movement has been recommended to recover motor function in patients after stroke.^[17]^ Children with hemiplegia should be encouraged to play with a ball or a ringing toy using both hands. Regarding the upper limb function, the unaffected side is usually likely to be the dominant hand, and in this case, the non-paralyzed left side performed elaborate movements, and the right hand was used as an auxiliary hand. The Enjoji Infantile Developmental Scale and Kyoto Scale of Psychological Development are common assessment scales for development used in Japan. If developmental delay in these screening tests is observed in preschool children, it might be desirable to refer them to the nursing period for school enrollment.

## 5 Conclusion

With an appropriate stepwise rehabilitation approach, the patient with hemiplegia due to AESD was able to make considerable recovery from paralysis and showed improvement in multiple functional aspects.

## Acknowledgments

We are deeply grateful to Dr Tomoko Yamaguchi (Department of Physical Medicine and Rehabilitation, Kanazawa University Hospital, Kanazawa, Japan) for her enthusiastic guidance on the rehabilitation of children and for the useful discussion.

## Author contributions

**Project administration:** Ai Takahashi, Erina Kamei, Yuri Sato, Seiichiro Shimada.

**Supervision:** Misao Tsubokawa, Akihiko Matsumine.

**Validation:** Genrei Ohta, Yusei Ohshima.

**Writing – original draft:** Ai Takahashi.

**Writing – review & editing:** Akihiko Matsumine.

## References

[1] Japan Intractable Diseases Information Center. Available at: https://www.nanbyou.or.jp/entry/4514

[2] Hoshino A Saitoh M Oka A. Epidemiology of acute encephalopathy in Japan, with emphasis on the association of viruses and syndromes. Brain Dev 2012;34:337–43. 21924570 10.1016/j.braindev.2011.07.012

[3] Mizuguchi M Ichiyama T Imataka G. Guidelines for the diagnosis and treatment of acute encephalopathy in childhood. Brain Dev 2021;43:02–31. 10.1016/j.braindev.2020.08.001 32829972

[4] Takanashi J Oba H Barkovich AJ. Diffusion MRI abnormalities after prolonged febrile seizures with encephalopathy. Neurology 2006;66:1304–9. 16682659 10.1212/01.wnl.0000210487.36667.a5

[5] Tada H Takanashi J Okuno H. Predictive score for early diagnosis of acute encephalopathy with biphasic seizures and late reduced diffusion (AESD). J Neurol Sci 2015;358:62–5. 26333951 10.1016/j.jns.2015.08.016

[6] Auvin S Bellavoine V Merdariu D. Hemiconvulsion-hemiplegia-epilepsy syndrome: current understandings. Eur J Paediatr Neurol 2012;16:413–21. 22341151 10.1016/j.ejpn.2012.01.007

[7] House JH Gwathmey FW Fidler MO. A dynamic approach to the thumb-in-palm deformity in cerebral palsy: Evaluation and results in fifty-six patients. J Bone Joint Surg Am 1981;63:216–25. 7462278

[8] Keio University Press Inc, Enjoji M Aiya T Kurikawa T. Enjoji Analytic Developmental Schedule for Infants (in Japanese). 1977.

[9] Kyoto International Social Welfare Exchange Centre; 2001;Ikuzawa Y Matsushita Nagase A. The Guide of Kyoto Scale of Psychological Development (in Japanese).

[10] Maegaki Y Kondo A Okamoto R. Clinical characteristics of acute encephalopathy of obscure origin: a biphasic clinical course is a common feature. Neuropediatrics 2006;37:269–77. 17236105 10.1055/s-2006-955928

[11] Okamoto R Fujii S Inoue T. Biphasic clinical course and early white matter abnormalities may be indicators of neurological sequelae after status epilepticus in children. Neuropediatrics 2006;37:32–41. 16541366 10.1055/s-2006-923949

[12] Fiser DH. Assessing the outcome of pediatric intensive care. J Pediatr 1992;121:68–74. 1625096 10.1016/s0022-3476(05)82544-2

[13] Palisano R Rosenbaum P Walter S. Development and reliability of a system to classify gross motor function in children with cerebral palsy. Dev Med Child Neurol 1997;39:214–23. 9183258 10.1111/j.1469-8749.1997.tb07414.x

[14] Kim CT Han J Kim H. Pediatric stroke recovery: a descriptive analysis. Arch Phys Med Rehabil 2009;90:657–62. 19345783 10.1016/j.apmr.2008.10.016

[15] Kozuka J Uno A Matsuda H. Relationship between the change of language symptoms and the change of regional cerebral blood flow in the recovery process of two children with acquired aphasia. Brain Dev 2017;39:493–505. 28159458 10.1016/j.braindev.2017.01.002

[16] Taub E Miller NE Novack TA. Technique to improve chronic motor deficit after stroke. Arch Phys Med Rehabil 1993;74:347–54. 8466415

[17] Han KJ Kim JY. The effects of bilateral movement training on upper limb function in chronic stroke patients. J Phys Ther Sci 2016;28:2299–302. 27630418 10.1589/jpts.28.2299 PMC5011582

